# The Effect of Wet Milling and Cryogenic Milling on the Structure and Physicochemical Properties of Wheat Bran

**DOI:** 10.3390/foods9121755

**Published:** 2020-11-27

**Authors:** Yamina De Bondt, Inge Liberloo, Chiara Roye, Erich J. Windhab, Lisa Lamothe, Roberto King, Christophe M. Courtin

**Affiliations:** 1 Laboratory of Food Chemistry and Biochemistry and Leuven Food Science and Nutrition Research Centre (LFoRCe), KU Leuven, Kasteelpark Arenberg 20, B-3001 Leuven, Belgium; inge.liberloo@kuleuven.be (I.L.); chiara.roye@kuleuven.be (C.R.); christophe.courtin@kuleuven.be (C.M.C.); 2 Food Process Engineering Group, ETH Zürich, LFO E18, Schmelzbergstrasse 9, 8092 Zürich, Switzerland; erich.windhab@hest.ethz.ch; 3 Institute of Materials Science, Nestlé Research, Route du Jorat 57, CH-1000 Lausanne, Switzerland; lisa.lamothe@rdls.nestle.com (L.L.); rokisol@hotmail.com (R.K.)

**Keywords:** wheat bran, particle size reduction, wet milling, cryogenic milling, water binding

## Abstract

Wheat bran consumption is associated with several health benefits, but its incorporation into food products remains low because of sensory and technofunctional issues. Besides, its full beneficial potential is probably not achieved because of its recalcitrant nature and inaccessible structure. Particle size reduction can affect both technofunctional and nutrition-related properties. Therefore, in this study, wet milling and cryogenic milling, two techniques that showed potential for extreme particle size reduction, were used. The effect of the milling techniques, performed on laboratory and large scale, was evaluated on the structure and physicochemical properties of wheat bran. With a median particle size (d_50_) of 6 µm, the smallest particle size was achieved with cryogenic milling on a laboratory scale. Cryogenic milling on a large scale and wet milling on laboratory and large scale resulted in a particle size reduction to a d_50_ of 28–38 µm. In the milled samples, the wheat bran structure was broken down, and almost all cells were opened. Wet milling on laboratory and large scale resulted in bran with a more porous structure, a larger surface area and a higher capacity for binding water compared to cryogenic milling on a large scale. The extensive particle size reduction by cryogenic milling on a laboratory scale resulted in wheat bran with the highest surface area and strong water retention capacity. Endogenous enzyme activity and mechanical breakdown during the different milling procedures resulted in different extents of breakdown of starch, sucrose, β-glucan, arabinoxylan and phytate. Therefore, the diverse impact of the milling techniques on the physicochemical properties of wheat bran could be used to target different technofunctional and health-related properties.

## 1. Introduction

Nowadays, there is an increasing demand from consumers for more healthy and qualitative food products, often with a focus on dietary fibre. Sufficient consumption of dietary fibre is associated with a reduced risk of several chronic diseases [1,2,3,4]. Wheat (*Triticum* *aestivum* L.) bran, a by-product of the production process of white flour, is known to be a good source of dietary fibre and other bioactive components, like phytochemicals, vitamins and minerals. The wheat bran milling fraction consists of the multilayered botanical bran structure consisting of a pericarp, testa and nucellar epidermis, together with the aleurone layer and remnants of the starchy endosperm [5]. The pericarp, the outer layer, is a rigid structure mainly consisting of empty cells made up of mostly arabinoxylan (AX), cellulose and lignin. The cubic shaped aleurone layer is characterised by the highest concentration of bioactive compounds, minerals and vitamins [6].

Although wheat bran has a high nutritional value, still a lot of refined cereal products are consumed. Between 2013 and 2016, whole grains accounted for only 15.8% of total grains intake among adults in the USA [7]. This can be attributed to the negative impact of bran addition on the processability and quality of grain-based products, like bread and biscuits [8,9]. To reduce these technofunctional drawbacks, different bran modification strategies like particle size reduction and (hydro)thermal or enzymatic treatments are already described in the literature [8]. However, conflicting observations are reported on the effect of particle size on final product quality, for example, bread quality. Campbell et al. [10] and Noort et al. [11] concluded that the negative influence of wheat bran on bread volume was enhanced when particle size was reduced until a median particle size of 520 µm and 50 µm, respectively. However, Moder et al. [12] observed an increase in bread volume for finely ground bran (97% passed a 150 µm sieve) compared to coarse bran, while Zhang and Moore [13] observed the highest bread volume for medium-sized bran (d_av_ = 415 µm) in comparison with coarse (d_av_ = 609 µm) and fine (d_av_ = 278 µm) wheat bran. They also indicated that addition of wheat bran with a small particle size resulted in bread with a lighter crust colour, better crust appearance and less gritty mouthfeel than bran with coarse or medium particle sizes. Jacobs et al. [14] concluded that conflicting observations reported in the literature could be attributed to the fact that water absorption and mixing time in the bread-making procedures were not optimised. They concluded that the particle size of wheat bran does not influence bread volume if optimal bread-making conditions are used. The detrimental technological effect of wheat bran can be, at least partly, explained by its hydration properties [8]. It has been shown that particle size reduction can have a significant effect on the hydration properties of wheat bran. Jacobs et al. [15] made a distinction between the total water retention capacity (TWRC) and the strong water retention capacity (SWRC) of wheat bran. The SWRC comprises the water that is bound in nanopores and through hydrogen bonding, while the TWRC also includes water bound in micropores and stacking water. The TWRC decreases in function of particle size reduction, while the SWRC stays constant until a particle size of 77 µm. However, going further down in particle size reduction with microfluidisation until 15 µm, De Bondt et al. [16] observed an increase in SWRC.

**Figure 1:**
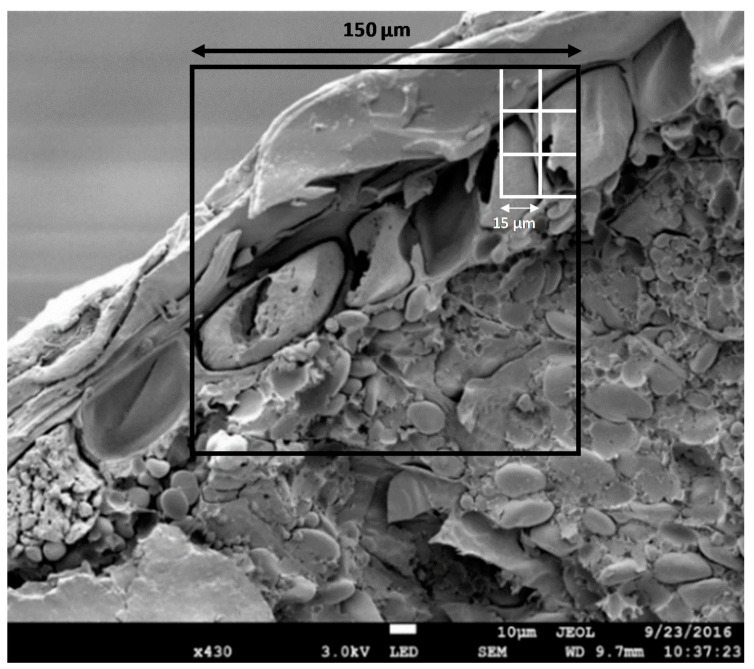
Cryo-SEM image of coarse wheat bran with a schematic indication of a square with a size of 150 µm, which is mostly the limit in particle size reduction of wheat bran in the literature, and 15 µm which is the aimed bran particle size in this study. The image was taken as described in [33].

Next to altered technological and sensorial properties, there are already indications that particle size reduction of wheat bran has a positive impact on its nutrition-related properties and physiology-related properties. A lot of bioactive components, like phenolic acids, phytate, minerals, and vitamins, are known to be bound to aleurone cell walls or entrapped in high concentrations in the aleurone cells. Particle size reduction of wheat bran can increase the extractability of antioxidants, like phenolic acids and anthocyanins, and thus increase the antioxidant capacity [17,18]. Besides, the extractability of AX is proven to increase as a result of bran particle size reduction [15]. It is known that native water-extractable AX (WE-AX) is more readily fermented in the colon compared to water-unextractable AX (WU-AX) [19]. Phytic acid is, on the one hand, considered as an antinutrient. It chelates minerals like iron, calcium, magnesium and zinc, which can lower their bioavailability in the human body. On the other hand, phytic acid can have a potential health benefit by acting as an antioxidant. Release of phytic acid from the aleurone cells in wheat bran by particle size reduction could increase the antioxidant capacity but decrease mineral bioavailability. However, complete breakdown of phytic acid could decrease the antioxidant capacity and improve mineral bioavailability [20,21,22]. Furthermore, the opening of the aleurone cell walls during particle size reduction could increase the bioavailability of different other components (minerals, vitamins) [6,23]. The aleurone cells are known to have dimensions of 37–65 µm by 25–75 µm. Micronisation of wheat bran below a particle size of 50 µm could mean a complete opening of aleurone cells and breakdown of the layered pericarp structure (Figure 1) [24]. 

It is clear that the particle size reduction of wheat bran can have significant implications in food systems and on health. However, particle size reduction of wheat bran in the literature is mostly limited to 150 µm, which implies only a partial breakdown of the structure. To ensure a complete breakdown of the wheat bran structure, and possibly improved nutrition-related effects and reduced technological drawbacks, particles below 50 µm should be obtained. Therefore, the objective of this study was to micronise wheat bran until a particle size of around 15 µm to ensure that all cells were broken down (Figure 1). There are indications that both wet and cryogenic milling can enhance the efficiency of the milling process and result in small (<50 µm), homogeneous particles [25,26,27,28]. Hemery et al. [25] stated that the moisture content and temperature of wheat bran has an impact on its mechanical properties. Increasing moisture contents of the bran increase its elasticity and plasticity [29], while at temperatures below the glass transition of the bran intermediate layer (−46 °C) (inner pericarp, testa, and nucellar tissue) wheat bran loses its plasticity [25]. Wet grinding of wheat bran was already shown to reduce the particle size more efficiently compared to dry grinding, with at the same time more homogeneous particle size distributions [26,27]. Yang et al. [27] observed that smaller wheat bran particles could be produced using wet ball milling (d_50_ = 10 µm) instead of dry ball milling (d_50_ = 91 µm) for 5 h. Milling of wheat bran at cryogenic temperatures favours a fast particle size reduction compared to milling at ambient temperatures [28]. Cryogenic milling is already frequently used in the pharmaceutical industry and for spices to better preserve volatile components [30,31,32].

However, a comprehensive study that simultaneously investigates the structure, hydration properties and chemical composition of wheat bran after wet and cryogenic milling is lacking. Therefore, in this study, we aimed to micronise wheat bran to an average particle size of around 15 µm with cryogenic and wet milling on laboratory and large scale. Insight was gained on how these techniques influence the wheat bran physicochemical properties and, therefore, could possibly impact food systems and health.

## 2. Materials and Methods 

### 2.1. Materials

Commercial coarse wheat (*Triticum aestivum* L.) bran was provided by Dossche Mills (Deinze, Belgium). The wheat bran was fully characterised by Roye et al. [33] and contains 11.8% starch, 20.9% proteins, 25.0% AX, 2.1% β-glucan, 3.3% fructan, 9.1% cellulose, 4.3% phytate, 5.4% lipids and 6.4% ash expressed on dry matter (dm) basis. All used chemical products were of analytical quality and purchased from Sigma-Aldrich (Bornem, Belgium) unless stated otherwise.

### 2.2. Wheat Bran Processing

The particle size of wheat bran was reduced using wet milling and cryogenic milling, performed on laboratory and pilot scale. 

#### 2.2.1. Wet Milling

Wet milling on a laboratory scale was performed with a wet bead mill (PML 2 Centex S2, Bühler, Uzwill, Switzerland). To prevent blockage of the sieve in the wet mill, wheat bran was premilled with a centrifugal mill (ZM200, Retsch GmbH, Haan, Germany). Afterwards, 0.8 kg of premilled bran was suspended in 3.2 L tap water in a vessel and pumped through the mill with a hose pump. This lab-scale mill had a throughput of 80 kg suspension per hour. Stainless steel beads of 1.6 mm (type Draison YUP) were kept in motion with a disc agitator (2700 rpm) and kept in the mill by a 0.8 mm screen. The milling chamber of this mill had a volume of 0.6 L and was filled for 75% of its volume by beads. The wheat bran was milled for 150 min, and samples were taken on different time points to be able to follow the particle size reduction in function of time. After 17, 36, 53, 70, 79, 88, 123 and 150 min of milling samples were taken. The samples were stored in the freezer (−25 °C) and after freeze-drying, the samples were pulverised with a blender to make sure all particles would pass a sieve with size 500 µm. The milling step was performed once, and characterisation measurements were done in triplicate. 

The wet bead milling process at a large scale was performed using a Cenomic 1 device (Bühler, Uzwill, Switzerland). Similar to the lab-scale process, a premilling step was done with a centrifugal mill (ZM200) to prevent blockage of the sieve. Preliminary experiments pointed out that a bead size of 1.6 mm, a bead filling of 75% and a concentration of bran in water of 10% were optimal for the large scale milling process. A diaphragm pump forced the suspension through the milling device with a milling chamber of 10.6 L. Zirconia beads (type Draison^®^ CP, CeriumPower, Bühler, Uzwill, Switzerland) with a diameter of 1.6 mm were used and kept in motion with a disc (1994 rpm). This wet mill had a throughput of 650 kg suspension per hour. Batches of 50 kg (10% bran) were milled for 6 h, and samples were taken every 20 min. The samples were stored in the freezer, and after freeze-drying, the samples were pulverised with a blender to make sure all particles would pass a sieve with size 500 µm. Milling was done in duplicate and characterisation of both samples was done in triplicate.

#### 2.2.2. Cryogenic Milling 

Cryogenic milling at laboratory scale was done with a cryogenic ball mill (Cryomill, Retsch GmbH). Two g of wheat bran was brought into a 50 mL milling chamber with 10 mm stainless steel beads (12 beads). The sample was cooled with liquid nitrogen (−196 °C) before and during milling. To make sure the wheat bran was completely frozen before milling, a precooling step was induced while the mill was oscillating at 300 rpm. Afterwards, the wheat bran was milled for 0, 2, 15, 30 and 45 min at 1800 rpm. Milling for 30 and 45 min was done in milling cycles of 15 min (1800 rpm) with an intermediate cooling step of 5 min at 300 rpm. Milling was done once and characterisation in triplicate. 

Large-scale cryogenic milling was done with an industrially available impact whirl mill. The DemiNo^®^ 2250 mill (Noll, Bobingen, Germany) worked under cryogenic conditions at a speed of 9200 rpm and has a capacity of up to 1000 kg per hour. The wheat bran was fed into a cryogenic screw (15 rpm) to cool the material with liquid nitrogen (−196 °C) before milling. The material had a temperature of −140 °C at the entry of the mill. After milling, a cyclone separated the dust particles from the bran. The outlet temperature of the material was −80 °C. The material was passed through the milling device two times, and samples were taken after each pass. Milling was done in duplicate and characterisation of both samples was done in triplicate.

### 2.3. Physical Characterisation of Wheat Bran

#### 2.3.1. Particle Size Analysis

The particle size of the coarse wheat bran was measured by sieving 20.0 g of bran on a set of sieves with mesh size 2000, 1000, 710, 500, 400, 250 and 50 µm. The sieves were shaken on a Vibratory Sieve Shaker (Retsch GmbH) for 20 min. The remaining mass on each sieve was weighed and used to calculate the cumulative particle size distribution. The mass-based median particle size (d_50_) is the particle size at which 50% of the material was sieved off and was determined by linear interpolation. Similarly, the particle sizes at which 10% (d_10_) or 90% (d_90_) of particles were sieved off were calculated. 

The particle size distribution of the milled wheat bran samples was measured by static light scattering. Approximately 50 mg of bran was suspended in 20 mL water and sonicated (20 kHz, amplitude 40%) for 1 min to disperse aggregates. The sample was added to the wet module of a laser diffraction Coulter LS320 particle size analyser (Beckman Coulter, Suarlée, Belgium) and analysed in quadruplicate. The particle size was calculated based on the Fraunhofer theory. 

#### 2.3.2. Microscopy 

Both light and fluorescence microscopy were used to visualise the structure of the (milled) wheat bran. Wheat bran samples were enclosed in 2% agar and fixed with Na-K phosphate buffer (100 mM, pH 7.0) with 3.0% paraformaldehyde and 1.0% glutaraldehyde. Afterwards, the samples were dehydrated with ethanol solutions with increasing ethanol concentrations (50, 70 and 95% ethanol) for at least two times 60 min and shaken overnight in absolute ethanol. The samples were infiltrated with resin using the Leica Historesin Embedding Kit and fixed with the Leica Historesin mounting medium (Leica Biosystems, Heidelberg, Germany). 

Cuts of the embedded samples with a thickness of 5 µm were made with a Leica RM 2255 microtome (Leica Biosystems) using a steel knife. The samples were incubated with Lugol (0.1% *w*/*v*), Light Green (0.1% *w*/*v*) and Calcofluor (0.01% *w*/*v*) for respectively 4, 4 and 3 min with intermediate rinsing. In this way, starch was visualised in purple, proteins in green and β-glucans in fluorescent blue. A Nikon ECLIPSE 80i epifluorescence microscope (Nikon Inc., Melville, New York, NY, USA) coupled with a Nikon Digital Sight DS-U2 camera was used for visualisation and data processed using the NIS elements software. The samples were visualised with light and fluorescence microscopy using a DAPI filter cube (excitation 325–375 nm, dichroic mirror 400 nm long pass, emission 435–485 nm). 

#### 2.3.3. Water Retention Capacity and Extractability

The total water retention capacity (TWRC) and extractability were determined by weighing 1.000 g of bran in a falcon tube, adding 30.0 mL of water, shaking for 30 min (150 rpm, room temperature) and decanting the supernatant after centrifugation (4000× *g*, 10 min, 25 °C). The pellet was weighed and reweighed after drying in an oven (90 °C) overnight. The TWRC and extractability were calculated by using the following formulas:

$$\mathrm{TWRC}\;\left(\frac{\mathrm{ml}}{g\;\mathrm{dm}}\right)=\;\frac{m_{\mathrm{pellet}\;\mathrm{before}\;\mathrm{drying}}-m_{\mathrm{pellet}\;\mathrm{after}\;\mathrm{drying}}}{m_{\mathrm{initial}\;\mathrm{sample},\mathrm{dry}}}$$

$$\mathrm{Extractability}\;\left(\%\right)=\;\frac{m_{\mathrm{initial}\;\mathrm{sample},\mathrm{dry}}-m_{\mathrm{pellet}\;\mathrm{after}\;\mathrm{drying}}}{m_{\mathrm{initial}\;\mathrm{sample},\mathrm{dry}}}\times 100$$

The strong water retention capacity (SWRC) was determined as described by De Bondt et al. [16], with small adaptations. 25 mg of sample was weighed on the upper part of a QIAprep Spin Miniprep Column (Qiagen, Hilden, Germany) and 350 µL of water was added. A column without sample was used as a blank to correct for the amount of water held by the filter. After one hour of resting, the samples were centrifuged (10,000× g, 10 min, 25 °C). The column was weighed and reweighed after drying in an oven (90 °C) overnight. Similar to the TWRC, the SWRC was calculated as the amount of water held by the bran, corrected for the blank.

#### 2.3.4. Nitrogen Physisorption

The specific surface area of the wheat bran samples was measured using a Meso 222 BET Surface Area Analyser (3P instruments, Odelzhausen, Germany) based on nitrogen physisorption at 77 K. Samples were freeze-dried, and approximately 1.000 g of sample was used for analysis. Degassing was done for 6 h at 60 °C, and the measurements were done at a dose amount of 1.0 mL/g until a relative pressure (P/P_0_) of 0.99.

### 2.4. Chemical Characterisation of the Aqueous Extracts of Wheat Bran

The moisture content of the (milled) wheat bran was determined according to AACC method 44-19.01 [34]. The carbohydrate composition, protein content and phosphorus content of the wheat bran extracts, made as described in Section 2.3.3, were determined as follows. 

Glucose, maltose, sucrose and fructose content were analysed with high-performance anion-exchange chromatography with pulsed amperometric detection (HPAEC-PAD) as described by Langenaeken et al. [35]. After addition of 100 µL internal standard (1.0 mg/mL rhamnose) to 1 mL of extract, samples were diluted 1:9 and filtered (0.22 µm). The samples were analysed on a ICS5000 chromatography system (Dionex, Sunnyvale, CA, USA) equipped with a CarboPac PA-100 (4 × 250 mm) column. The mobile phase consisted of 100mM NaOH during equilibration and during the first 5 min of the measurement. Afterwards, a sodium acetate gradient was applied over 25 min at a rate of 3.6 mM/min while keeping NaOH isocratic at 100 mM. The flow rate was set at 1 mL/min. A calibration solution was used to identify and quantify the different mono- and disaccharides in the wheat bran extracts.

The glucose content after hydrolysis of the extract and the WE-AX content were determined with gas chromatography based on the method described by Courtin et al. [36]. Carbohydrates in the wheat bran extract were hydrolysed to monosaccharides with 2.0 M trifluoroacetic acid, reduced to alditols with NaBH_4_ and derivatised to alditol acetates using acetic acid anhydride. The amount of glucose, arabinose and xylose was measured, and the WE-AX content was calculated as the sum of the arabinose and xylose levels multiplied by 0.88 to account for its polymeric nature. 

The water-extractable β-glucan content was determined using the Megazyme procedure for mixed-linkage β-glucan in fibre samples. Approximately 50.0 mg of freeze-dried wheat bran extract was used for the analysis. 

The water-extractable protein content was determined on freeze-dried wheat bran extract using an automated micro-Dumas protein analysis system (Carlo Erba EA1108, Milano, Italy). An adapted version of the AOAC method 990.03 was applied [37], and a conversion factor of 6.25 was used. 

The free and total phosphorus content of wheat bran aqueous extract and of wheat bran were determined with the Phytic Acid Assay kit of Megazyme, with adaptations. Wheat bran aqueous extracts were made as described in 2.3.3 and used as such. For the measurement on wheat bran, 0.5 g of wheat bran was extracted with 50 mL of 0.66 M HCl for 16 h while shaking (150 rpm) and afterwards neutralised with NaOH (0.75 M). The free phosphorus was measured colourimetrically (λ = 655 nm) using ammonium molybdate and a phosphorus calibration curve. For determination of the total phosphorus content, the sample was first treated with a phytase and alkaline phosphatase to release the phosphorus from inositol phosphates. Afterwards the total phosphorus content was determined colourimetrically. The ratio of free on total phosphorus content was calculated to give an idea about the amount of phosphorus bound to inositol phosphates. 

### 2.5. Statistical Analyses

Significant differences were detected by performing one-way ANOVA using JMP Pro 14 (SAS Institute, Cary, NC, USA), with a comparison of mean values using the Tukey test (α < 0.05). Triplicate measurements after lab-scale milling and the average of the triplicate measurements after large scale milling for two times were used.

## 3. Results and Discussion

### 3.1. Effect of Wet and Cryogenic Milling on the Particle Size Distribution of Wheat Bran

Coarse wheat bran had a particle size distribution with a d_10_, d_50_ and d_90_ of 708, 1298 and 1835 µm, respectively. The particle size of wheat bran was reduced with wet milling and cryogenic milling, both on laboratory and pilot scale. 

**Figure 2:**
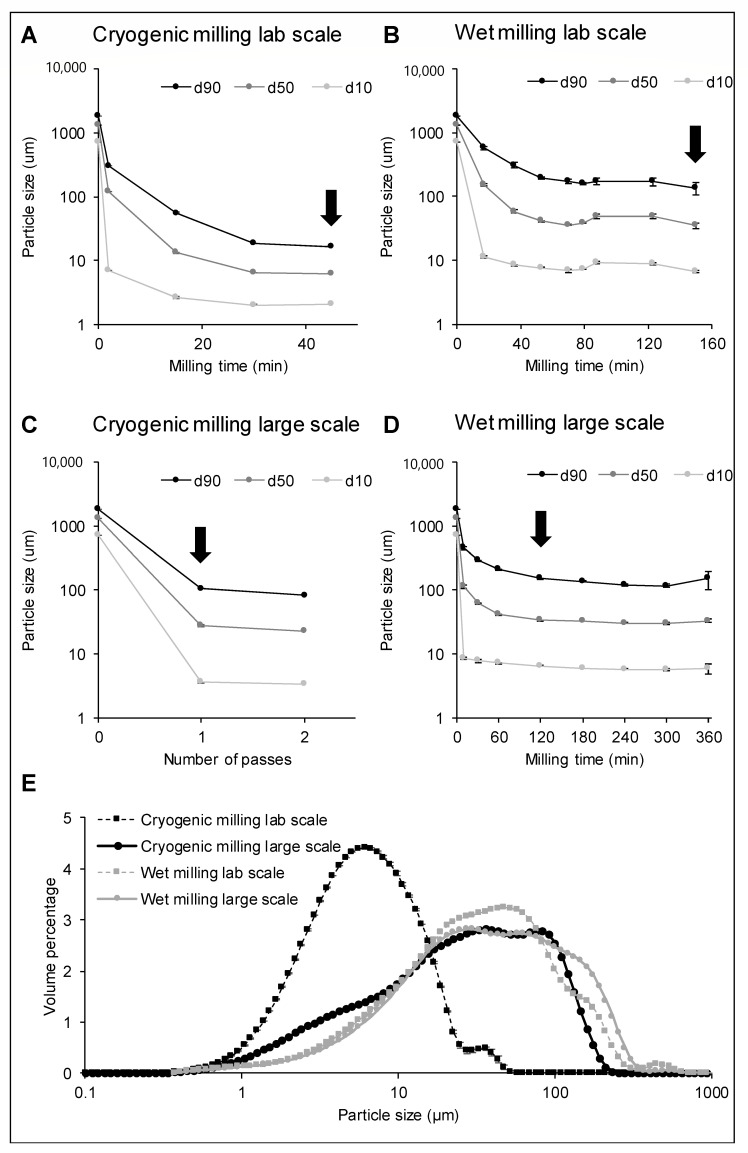
The particle size distribution of wheat bran as a function of time or number of passes during (**A**) cryogenic milling on a laboratory scale, (**B**) wet milling on a laboratory scale, (**C**) cryogenic milling on a large scale, (**D**) wet milling on a large scale. d10, d50 and d90 correspond to the diameter where 10, 50 or 90% of the distribution has a smaller particle size. The samples that were further characterised are indicated with arrows in (**A**–**D**), and their particle size distribution in percentage by volume is shown in (**E**).

The evolution of the particle size in function of the milling time for cryogenic and wet milling on a laboratory scale can be found in Figure 2A,B. The d_50_ of the bran reached a plateau at 6 µm after 30 min of cryogenic milling. With wet milling on a laboratory scale, particle size reduction was slower, and the d_50_ only reached a plateau at 40 µm after 53 min of milling. This trend was also reflected in the d_90_, meaning that the proportion of large particles still decreased until, respectively, 30 and 53 min, after which a plateau was reached, and 90% of the particles were below a particle size of 19 µm and 195 µm. For both milling techniques, a homogenous unimodal particle size distribution was obtained (Figure 2E). 

Upscaling of cryogenic milling was performed with an impact mill instead of a ball mill as this was industrially available and Hemery et al. [28] proved that the technique was of minor importance for particle size reduction with cryogenic grinding. Wheat bran was passed twice through the cryogenic mill, and after each pass, the particle size was measured (Figure 2C). A large particle size reduction was obtained after the first pass (d_50_ of 28µm), but the particle size reduction in a successive milling cycle was limited. The plateau of the particle size was higher for cryogenic milling on a large scale than on laboratory scale. For wet milling on a large scale, the same type of equipment was used as on laboratory scale, being a bead mill with beads of 1.6 mm. The particle size in function of the milling time is shown in Figure 2D. A similar trend was seen for the large-scale wet milling process as for the lab-scale wet milling. After 120 min of wet milling on a large scale, a plateau was reached with a d_50_ around 30 µm. For each milling technique, one sample was chosen for further characterisation, as is indicated in Figure 2 with arrows. For the laboratory scale milling processes, the samples at the largest milling times were chosen to examine the effect of the highest impact on the wheat bran. For the large scale milling, a sample after one pass through the cryogenic mill and a sample after 120 min in the large scale wet mill were chosen as no further particle size reduction was taking place and preliminary screening of the samples in function of time indicated no further changes in physical properties. Figure 2E shows the particle size distribution as a percentage by volume for these samples.

The smallest wheat bran particles were produced using milling under cryogenic conditions on a laboratory scale (d_50_ of 6 µm after 30 min of milling). The effectiveness of cryogenic milling was already indicated by Hemery et al. [28], who stated that temperatures below the glass transition of bran assist a fast particle size reduction and a homogenous and narrow particle size distribution. Below the glass transition temperature of the intermediate layer (−46 °C), the bran material is more brittle. Hemery et al. [28] observed a d_50_ of 55 µm and 33 µm after one and two passes through a cryogenic impact mill respectively, which was similar to our results with a large scale cryogenic impact mill. However, with the cryogenic ball mill on a laboratory scale, a significantly larger particle size reduction could be achieved (d_50_ of 6 µm). In this sample, 90% of the particles were smaller than 17 µm, which is to the best of our knowledge the smallest particle size of wheat bran reported in the literature.

Yang et al. [27] observed that by wet ball milling for 5 h, wheat bran particles with a median particle size of 10 µm could be produced. This was not reflected in our results, where after six hours of large-scale wet milling a median particle size of 33 µm was reached. The same particle size limit was seen for lab-scale milling. After a certain timepoint, there was no considerable difference in particle size in function of milling time anymore. This time point was different for the laboratory scale (53 min) and large scale (120 min) mill, so dependent on the equipment and scale.

### 3.2. Effect of Wet and Cryogenic Milling on the Structure of Wheat Bran 

**Figure 3:**
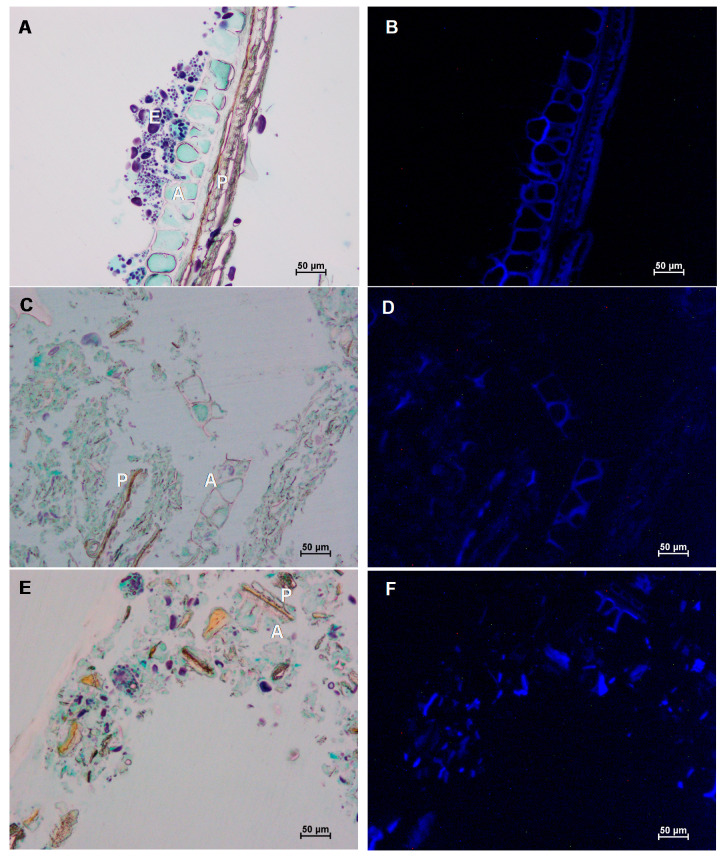
Visualisation of untreated coarse wheat bran (**A**,**B**) and wheat bran that was cryogenically milled (**C**,**D**) or wet milled (**E**,**F**) on a large scale. In the milled samples almost all bran structures were broken down but in these pictures we focused on a few partially intact structures. Samples were stained with Lugol, Light Green and Calcofluor and visualised with light microscopy (**A**,**C**,**E**) and fluorescence microscopy (**B**,**D**,**F**). The pericarp (P), aleurone (**A**) and residual endosperm (**E**) are indicated.

**Figure A1:**
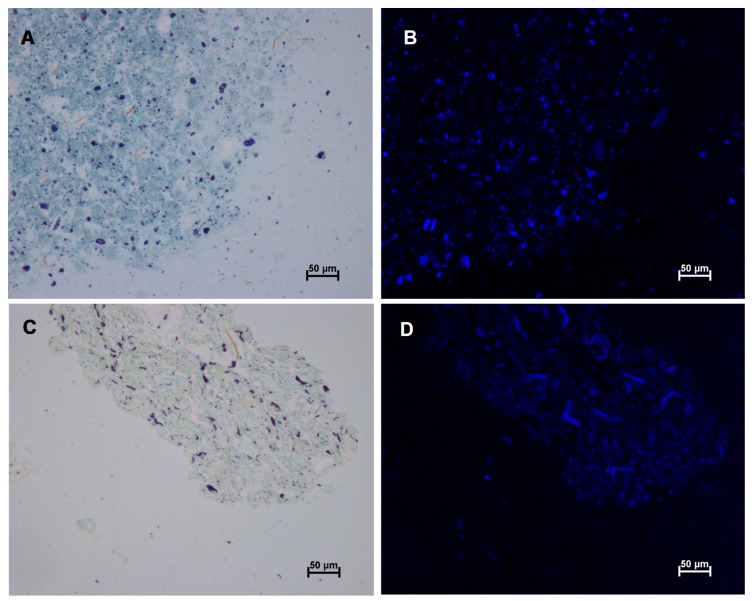
Visualisation of wheat bran that was cryogenically milled (**A**,**B**) or wet milled (**C**,**D**) on a laboratory scale. Samples were stained with Lugol, Light green and Calcofluor and visualised with light microscopy (**A**,**C**) and fluorescence microscopy (**B**,**D**).

The structure of wet and cryogenically milled wheat bran was visualised using light and fluorescence microscopy (Figure 3 and Figure A1 in Appendix A). 

For wet and cryogenic milling, both on laboratory and pilot scale, one milling condition was chosen as indicated in Figure 2 with arrows. The untreated coarse wheat bran consists of a multilayered structure composed of a pericarp, an intermediate layer and aleurone layer with, attached to it, residual endosperm. After wet or cryogenic milling on laboratory and large scale, almost all bran structures were broken down (<50 µm). When we searched for a few partially intact structures and focused on them during microscopy, a clear difference between wet and cryogenic milling could be observed (Figure 3C,E). During wet milling, the dissociation of the bran layers and separate aleurone and pericarp layers could be observed. Hemery et al. [25] observed that at temperatures between −10 °C and 25 °C, the pericarp breaks before the other layers and at a different location, which makes it possible to dissociate the pericarp from the other layers. Cryogenic milling, in contrast, facilitated a simultaneous breakage of the different bran layers. The fact that no dissociation of the bran layers took place during cryogenic milling was already reported in the literature and can be explained by the fact that the intermediate layer is in the glass state at temperatures below −46 °C [25,28]. 

### 3.3. Effect of Wet and Cryogenic Milling on the Physical Properties of Wheat Bran

**Figure 4:**
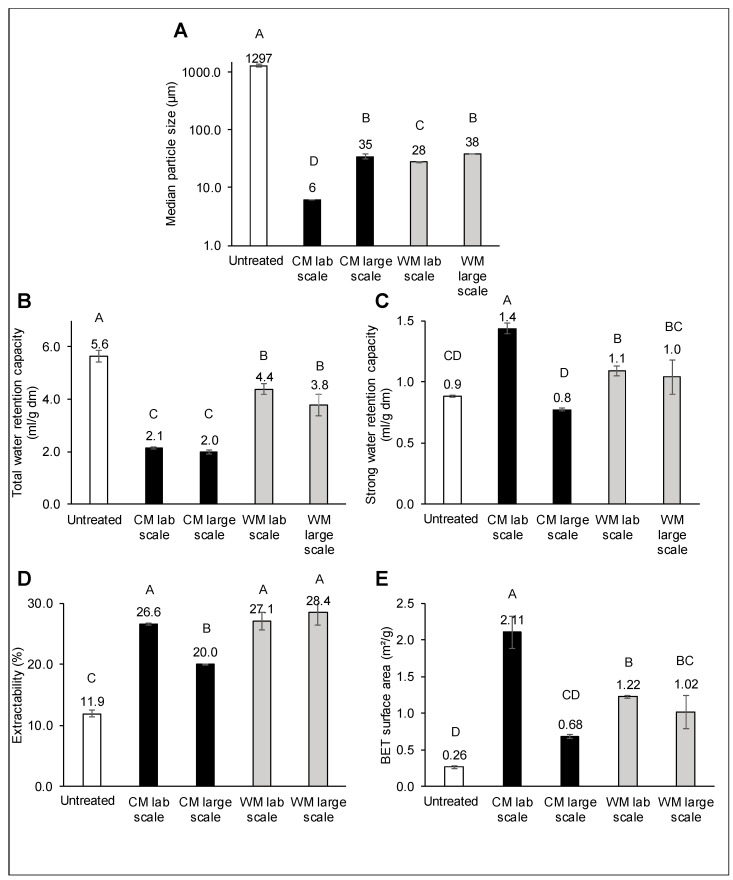
Physical properties of untreated coarse wheat bran and wheat bran that was cryogenically (CM) or wet (WM) milled on a laboratory or large scale. (**A**) Particle size, (**B**) Total and (**C**) Strong water retention capacity, (**D**) Extractability and (**E**) BET surface area. Means with different letters are significantly different (*p* < 0.05).

In Figure 4, the physical properties of the different selected wheat bran samples are presented. The TWRC of wheat bran is an important parameter because it can give an indication of its physiology-related and technological properties. A higher TWRC of (modified) wheat bran is related to an increased bulking effect [38]. Moreover, wheat bran addition has an impact on the water distribution of food products [8]. 

Untreated coarse wheat bran had a TWRC of 5.6 mL/g dm. Wet milling and cryogenic milling decreased the TWRC to 3.8–4.4 mL/g dm and 2.0–2.1 mL/g dm. There was no significant effect of the scale of milling (Figure 4B). Jacobs et al. [15] observed that the TWRC of wheat bran decreased in function of particle size from 6 mL/g dm for coarse bran until around 3 mL/g dm for wheat bran with an average particle size of 77 µm. This was explained by the breakdown of micropores and the loss of stacking water in between particles. Although cryogenic milling on a large scale and wet milling on laboratory and large scale resulted in similar particle sizes, the TWRC for wet milling was significantly higher than for cryogenic milling. This could indicate that a more porous structure is formed during wet milling and subsequent freeze-drying. Besides, the lab-scale cryogenically milled bran had a much smaller particle size than the large-scale milled bran, but no effect on TWRC could be observed. To further investigate these differences, the SWRC was determined (Figure 4). 

The SWRC of untreated coarse wheat bran (0.9 mL/g dm) slightly increased during wet milling to 1.0–1.1 mL/g dm. However, cryogenic milling on a laboratory scale increased the SWRC to 1.4 mL/g dm, while cryogenic milling on a large scale had no effect (0.8 mL/g dm). Jacobs et al. [15] indicated that dry milling until an average particle size of 77 µm did not affect the SWRC, while De Bondt et al. [16] observed an increase in SWRC until 1.8 mL/g dm upon particle size reduction by microfluidisation to 15 µm. We can, therefore, say that both the method for particle size reduction and the achieved particle size have an effect on the SWRC. The SWRC can be attributed to water bound in cell wall nanopores and through hydrogen bonds. Therefore, the BET surface area of the different samples was determined (Figure 4E). Compared to the specific surface area of coarse wheat bran (0.26 m²/g), cryogenic milling on a laboratory scale resulted in the most substantial increase (2.11 m²/g). Cryogenic milling on a large scale only resulted in an increase in specific surface area up to 0.68 m²/g. This indicates that the difference in SWRC could be linked to the specific surface area, and more water could be bound in nanopores or through hydrogen bonds after laboratory-scale cryogenic milling. These differences could be linked to the different particle size obtained after cryogenic milling on laboratory and large scale. If we assume that after laboratory and large-scale cryogenic milling, spheres with the same density are formed, we would expect that particles with a size of 6 µm have 5.6 times higher specific surface area than particles with size 35 µm. We observed 3 times higher specific surface area only, so this indicates that the assumptions are not entirely correct, but it shows the possible effect of changes in particle size. However, while wet milling resulted in similar particle size as cryogenic milling on a large scale, the BET surface area of the wet-milled wheat bran is higher (1.02–1.22 m²/g) than the cryogenically milled wheat bran on a large scale (0.68 m^2^/g). The higher specific surface area and SWRC show that wet milling can result in a more accessible and porous structure. We hypothesise that during wet milling, there is a combined mechanical and enzymatic impact on the bran. In the wet environment of the wet milling procedure, the endogenous enzymes of the wheat bran, like xylanase and α-amylase, can cause degradation of structural components. This could possibly lead to a structure that is more accessible for hydrogen bonding with water. Furthermore, the freeze-drying process after wet milling could have an effect on the SWRC as freeze-drying could open the structure of the wheat bran and thereby increase its interaction with water. The higher extractability of the wet-milled bran compared to the cryogenically milled bran on a large scale also gives an indication that the structure is more accessible (Figure 4D). 

The aqueous extractability of wheat bran can give an indication of the physiology and nutrition-related characteristics and technofunctionality of the bran. The aqueous extractability increased from 11.9% for the coarse bran to 20.0–28.4% for the milled wheat bran. More opening of aleurone cells and breakdown of the structure led to an increased release of components in the extract. Cryogenically milled bran on a laboratory scale has a larger extractability than on a large scale, which can be linked to the difference in particle size. The high extractability for the wet-milled wheat bran is probably linked to the degradation of the structure by endogenous enzymes in the wet environment (Figure 4D). To further investigate this, the chemical composition of the aqueous extract of the different wheat bran samples was determined. 

### 3.4. Effect of Wet and Cryogenic Milling on the Chemical Composition of the Aqueous Extract of Wheat Bran 

To get more insight in the composition of the aqueous extract, the concentration of carbohydrates, proteins and phosphorus was measured (Table 1). 

The total amount of glucose after hydrolysis of the extract was higher in the wet-milled samples (18.53–19.26% dm) compared to the coarse bran (4.20% dm). The increase in extractability is, therefore, mainly coming from an increase in glucose content after hydrolysis. Cryogenic milling on a laboratory scale also slightly increased the total water-extractable glucose content (9.43%), but cryogenic milling on a large scale had no effect compared to coarse wheat bran. This again indicates that cryogenic milling on laboratory and large-scale results in different physicochemical properties. Milling at laboratory scale with a cryogenic ball mill apparently was more intensive than milling with an impact whirl mill on large scale. The total glucose content in the extract can originate from free glucose, maltose, maltodextrins, sucrose, β-glucan and fructan. During wet milling, starch attached to the wheat bran (11.8%) is degraded into free glucose (3.5–4.2%), maltose (4.5–4.7%) and maltodextrins (not measured) by amylases that are active in the wet milling environment. Wheat bran contains various enzymes like amylases, phytases, endoxylanases and lipases that are mainly accumulated in the aleurone layer or originate from microorganisms populating the wheat bran [8,39,40]. Breakdown of the aleurone layer can liberate endogenous enzymes, and the wet environment during wet milling will promote enzymatic activity. However, cryogenic milling on a laboratory scale also increases the maltose (3.07%) and maltodextrin content. This can indicate that starch is also broken down mechanically. The sucrose content was the largest in the wheat bran that was cryogenically milled on a laboratory scale. This indicates that in the other samples sucrose is degraded into fructose and glucose. During wet milling, all sucrose is degraded, which can indicate that, in this case, the endogenous enzymatic activity is also important. During cryogenic milling, no enzyme activity can take place, however during the extraction procedure enzymes can be active. It is, therefore, possible that the sucrose in coarse and large scale cryogenically milled wheat bran is enzymatically degraded during the extraction procedure. We hypothesise that during cryogenic milling on a laboratory scale, endogenous enzymes are inactivated because of the larger mechanical impact. Therefore no sucrose degradation took place during the extraction procedure. As initially there is at least 2.29% sucrose present in the wheat bran (cryogenic milling laboratory scale), sucrose degradation during wet milling can only account for 1.2% free fructose and 1.2% free glucose. This indicates that wet milling also degrades part of the fructan (3.3%) into free fructose. The extractability of β-glucan increased during wet milling and cryogenic milling on a laboratory scale. This can indicate that β-glucan becomes more accessible because of the structure breakdown or that water-unextractable β-glucan is converted into WE- β-glucan by depolymerisation. Besides carbohydrates built up from glucose, also WE-AX was measured in the extract. Milling increased the amount of WE-AX compared to coarse wheat bran from 0.53% to 1.01–2.84%. Particle size reduction is already known to convert WU-AX to WE-AX by the breakdown of the cell structures on macroscale and cleavage of the AX structure by impact forces on microscale [15,41]. We can conclude that wet milling drastically alters the carbohydrate composition of wheat bran extract, mostly because of enzymatic activity during the process. Cryogenic grinding on a large scale has only minor effects on the carbohydrate composition of the aqueous wheat bran extract. However, very intensive cryogenic grinding (on a laboratory scale) can also affect the carbohydrate composition of the extract mechanically. These changes can have a nutritional and technofunctional impact. For example, WE-AX is known to be more accessible for the microbiota in the colon and thus more fermentable compared to WU-AX [19]. Moreover, WU-AX and WE-AX have different functionality in food systems [42]. 

The amount of water-extractable proteins increased for both wet-milled (4.25–4.64%) as for cryogenically milled (5.35–6.50%) wheat bran compared to the coarse wheat bran (2.91%). This could be explained by the breakdown of the bran structure during milling and, more specifically, the opening of the aleurone cells, which have the highest concentration of proteins [6]. Wet milling resulted in a lower increase in extractable proteins compared to cryogenic milling, both on laboratory and large scale. Rosa-Sibakov et al. [26] attributed a lower amount of soluble proteins with wet grinding compared to dry grinding to the formation of protein aggregates by disulphide- or covalent bonds.

Phosphorus in wheat bran is stored as phytic acid (4.3%). It is considered as an antinutrient as it chelates minerals, which makes them less bioavailable in the human body [20]. The total phosphorus content of the wheat bran samples was determined after extraction with HCl. The coarse and cryogenically milled bran had a total phosphorus content of 1.03–1.11%, while 1.26–1.38% phosphorus was extracted from the wet-milled bran. This indicates that extraction with HCl does not extract all phosphorus from the coarse and cryogenically milled wheat bran. The extracted phosphorus in these samples is mainly present as phytate because the ratio of free on total phosphorus is only 0.08–0.09. It can, therefore, be assumed that no mechanical breakdown of phytic acid was occurring. The phosphorus in the wet-milled samples is, however, mostly present as free phosphorus as the ratio of free on total phosphorus is 0.78–0.79. This can indicate phytase activity during wet milling. Guo et al. [40] also found that phytic acid can be broken down by endogenous phytases already at room temperature. Breakdown of phytic acid and thus a higher ratio of free on total phosphorus could therefore increase the bio-accessibility of minerals in wheat bran [20,22]. Aqueous extraction of the wheat bran samples always resulted in less phosphorus than extraction with HCl. Cryogenic milling increased the WE-total phosphorus content from 0.33 to 0.67–0.72% (Table 1). After wet milling, almost all phosphorus became water-extractable (1.15%–1.19%). After aqueous extraction, the ratio of WE-free on WE-total phosphorus varied between 0.70–0.89, which indicates that, during the extraction procedure, phytic acid is broken down by endogenous phytase activity.

## 4. Conclusions

Overall, we can conclude that it is possible to change the structure and physicochemical properties of wheat bran in different ways with wet and cryogenic milling. The smallest particle size was achieved with cryogenic milling on a laboratory scale (d_50_ = 6 µm), while cryogenic milling on a large scale and wet milling on a laboratory and large scale resulted in a similar particle size reduction (d_50_ = 28–38 µm). With both techniques, it was possible to break down the multilayered wheat bran structure and make the aleurone cell content accessible. The mechanical impact of the cryogenic mill on a laboratory scale was very high. During wet milling, a combination of mechanical impact and enzymatic breakdown took place. Further research is needed to investigate the feasibility of milling techniques. While possible effects on the storage stability, drying and cooling costs should be taken into account, differences in hydration properties and chemical composition showed the potential of the milling techniques to target different bran properties that can affect the behaviour of wheat bran in food products or its physiological impact. The proposed hypotheses concerning the nutritional and physiological effects of the milling techniques should be verified. As an example, the increase in extractable dietary fibre content and the more accessible structure, measured as specific surface area, could lead to an increase in the short-chain fatty acid production upon fermentation in the colon. As a suggestion, an in vitro fermentation study or a human intervention study could be performed. Besides, wheat bran samples should be incorporated in food products, to link their physicochemical properties as characterized in this study, to their functionality in food products.pendix

## Figures and Tables

**Table 1:** Chemical composition of aqueous extracts from untreated coarse wheat bran and from wheat bran that was cryogenically or wet milled on laboratory or large scale. The measured components are expressed on the total dry mass (dm) of the wheat bran. Means with different letters are significantly different (*p* < 0.05).

		Cryogenic Milling		Wet Milling	
	No Treatment	Laboratory Scale	Large Scale	Laboratory Scale	Large Scale
Total glucose content after hydrolysis of extract (% dm)	4.20 (0.13) ^c^	9.43 (0.46) ^b^	4.44 (0.01) ^c^	18.53 (1.41) ^a^	19.26 (0.82) ^a^
Glucose (% dm)	0.58 (0.06) ^c^	0.37 (0.01) ^c^	0.87 (0.02) ^c^	4.24 (0.18) ^a^	3.52 (0.37) ^b^
Maltose (% dm)	0.06 (0.05) ^c^	3.07 (0.21) ^b^	0.17 (0.01)^c^	4.48 (0.21) ^a^	4.66 (0.41) ^a^
Sucrose (% dm)	1.87 (0.09) ^b^	2.29 (0.10) ^a^	1.64 (0.01) ^c^	ND	ND
WE-β-glucan (% dm)	0.14 (0.01) ^c^	0.96 (0.04) ^a^	0.28 (0.02) ^c^	0.64 (0.03) ^b^	0.61 (0.20) ^b^
Fructose (% dm)	0.46 (0.05) ^c^	0.21 (0.02) ^d^	0.66 (0.02) ^c^	2.70 (0.16) ^a^	2.20 (0.01) ^b^
WE-AX (% dm)	0.53 (0.02) ^d^	2.84 (0.16) ^a^	1.01 (0.01)^c^	1.78 (0.13) ^b^	1.84 (0.01) ^b^
WE-protein content (% dm)	2.91 (0.31) ^d^	5.35 (0.09) ^b^	6.50 (0.11) ^a^	4.25 (0.03) ^c^	4.64 (0.35) ^c^
WE-total phophorus (%dm)	0.33 (0.02) ^c^	0.67 (0.04) ^b^	0.72 (0.01) ^b^	1.15 (0.06) ^a^	1.19 (0.24) ^a^
$\frac{\mathrm{WE}-\mathrm{free}\;\mathrm{phosphorus}}{\mathrm{WE}-\mathrm{total}\;\mathrm{phosphorus}}$	0.83 (0.08) ^ab^	0.70 (0.05) ^b^	0.83 (0.00) ^ab^	0.89 (0.02) ^a^	0.89 (0.03) ^a^

ND = not detectable, WE = water-extractable, AX = arabinoxylan.
